# A vendor-agnostic, PACS integrated, and DICOM-compatible software-server pipeline for testing segmentation algorithms within the clinical radiology workflow

**DOI:** 10.3389/fmed.2023.1241570

**Published:** 2023-10-26

**Authors:** Lei Zhang, Wayne LaBelle, Mathias Unberath, Haomin Chen, Jiazhen Hu, Guang Li, David Dreizin

**Affiliations:** ^1^School of Medicine, University of Maryland, Baltimore, MD, United States; ^2^Department of Computer Science, Whiting School of Engineering, Johns Hopkins University, Baltimore, MD, United States

**Keywords:** artificial intelligence, deep learning, nnU-net, PACS, DICOM, OHIF, quantitative visualization, computed tomography

## Abstract

**Background:**

Reproducible approaches are needed to bring AI/ML for medical image analysis closer to the bedside. Investigators wishing to shadow test cross-sectional medical imaging segmentation algorithms on new studies in real-time will benefit from simple tools that integrate PACS with on-premises image processing, allowing visualization of DICOM-compatible segmentation results and volumetric data at the radiology workstation.

**Purpose:**

In this work, we develop and release a simple containerized and easily deployable pipeline for shadow testing of segmentation algorithms within the clinical workflow.

**Methods:**

Our end-to-end automated pipeline has two major components- 1. A router/listener and anonymizer and an OHIF web viewer backstopped by a DCM4CHEE DICOM query/retrieve archive deployed in the virtual infrastructure of our secure hospital intranet, and 2. An on-premises single GPU workstation host for DICOM/NIfTI conversion steps, and image processing. DICOM images are visualized in OHIF along with their segmentation masks and associated volumetry measurements (in mL) using DICOM SEG and structured report (SR) elements. Since nnU-net has emerged as a widely-used out-of-the-box method for training segmentation models with state-of-the-art performance, feasibility of our pipleine is demonstrated by recording clock times for a traumatic pelvic hematoma nnU-net model.

**Results:**

Mean total clock time from PACS send by user to completion of transfer to the DCM4CHEE query/retrieve archive was 5 min 32 s (± SD of 1 min 26 s). This compares favorably to the report turnaround times for whole-body CT exams, which often exceed 30 min, and illustrates feasibility in the clinical setting where quantitative results would be expected prior to report sign-off. Inference times accounted for most of the total clock time, ranging from 2 min 41 s to 8 min 27 s. All other virtual and on-premises host steps combined ranged from a minimum of 34 s to a maximum of 48 s.

**Conclusion:**

The software worked seamlessly with an existing PACS and could be used for deployment of DL models within the radiology workflow for prospective testing on newly scanned patients. Once configured, the pipeline is executed through one command using a single shell script. The code is made publicly available through an open-source license at “https://github.com/vastc/,” and includes a readme file providing pipeline config instructions for host names, series filter, other parameters, and citation instructions for this work.

## Introduction

### Unmet need for open-source software integrating DL models into quantitative visualization clinical workflows

Simple, reproducible approaches are needed to bring AI/ML for medical image analysis closer to the bedside. During the Radiologic Society of North America (RSNA) 2018 Artificial Intelligence (AI) Summit, researchers, opinion leaders, and early adopters of AI computer-aided detection/diagnosis (CAD) radiology innovations emphasized that frameworks are needed to integrate machine learning (ML) algorithms into clinical practice (1), but few open source solutions to this problem have been reported. For methods involving quantitative visualization (i.e., those that produce segmentation results with volumetric data), solutions are needed that present segmentations as Digital Imaging and Communications in Medicine (DICOM) SEG objects and volumetric measurements as DICOM structured report (SR) elements, since the DICOM format is required in clinical work. In the future, SR elements could be used to autopopulate radiology reports, provided that timely results can be generated prior to report completion.

The Radiologic Society of North America (RSNA) recently released a special report on clinical AI implementation and presented a road map for governance, including a framework for required infrastructure (2). Clinical artificial intelligence/machine learning (AI/ML) integration in the DICOM format is necessary for deployment of commercial vendor-specific quantitative CAD tools. But imaging departments may want to deploy and test locally developed algorithms as well, and this can be facilitated with open-source vendor agnostic methods (3). Such algorithms commonly employ research-grade code and models, are at the stage of preliminary testing and validation, use the Neuroimaging Informatics Technology Initiative (NIfTI) format as input and output, and are not ready for clinical use. However, researchers may want to evaluate generalizability on new cases as they arise in the clinical workflow or conduct prospective studies of diagnostic performance, prognostic utility, or user acceptance.

In radiology, criteria that need to be met for pre-clinical “shadow-mode” testing of AI/ML CAD tools include cross-platform and cross-domain integration, as well as data security and access. A vendor-agnostic platform should be integrated with hospital imaging archival systems. These pre-conditions are also critical for data search and retrieval in the pathology domain (4, 5). Jansen et al. (6) developed a vendor-agnostic EMPAIA (EcosysteM for Pathology Diagnostics with AI assistance) platform which is used for integrating AI applications into digital pathology infrastructures.

Within the radiology clinical workflow, the DICOM standard includes a large library of metadata to facilitate interoperability for storing and visualization in Picture Archiving and Communications Systems (PACS). Sohn et al. (7) released a vendor-agnostic PACS-compatible solution for integrating AI into the radiology workflow. They showed feasibility of their pipeline for 2D classification of breast density on mammograms. However, the method did not include functionality for visualizing DICOM SEG and SR elements necessary for quantitative imaging.

As AI/ML methods improve along the technology readiness pipeline, clinical-translational teams working on precision imaging solutions will be increasingly interested in deploying trained cross-sectional imaging-based models that segment and volumetrically quantify pathology for pre-clinical evaluation on new cases in “real world” settings as they arise (2, 8). Granular quantitative volumetric information can provide objective metrics for personalized decision-making and treatment planning in clinical workflows (8). Such quantitative visualization (QV) tools fall under FDA computer-aided diagnosis (CADx) or image processing and quantification (IPQ) Software as Medical Device (SaMD) designations (9).

### Necessary elements for a clinical workflow-compatible software server pipeline

Simple, modular, and open-source PACS integrated pipelines are needed that are tailored specifically for segmentation and quantitative visualization tasks applied to cross-sectional imaging modalities. DICOM lacks the elegant design features of the NIfTI format for cross-sectional medical image processing and analysis (10, 11). Conversely, PACS systems do not support data handling of NIfTI image volumes used as model input and output.

Each slice of a DICOM CT series is represented by a.dcm file, whereas the NIfTI series employed in segmentation algorithm development is represented as a volume in a single.nii.gz file. DICOM to NIfTI and NIfTI to DICOM conversion bridges the gap between clinical PACS and visualization for quantitative imaging (11). A listener and router are needed to handle the flow of data for file conversion, image processing, and viewing of coregistered segmentation masks and quantitative results. JSON files are needed to specify relevant DICOM metadata such as pathology type and display color for a given DICOM SEG (segmentation) object, and the DICOM SEG object needs to be associated with its original DICOM image series through a DICOM unique identifier (UID). The precise volume of pathology (e.g., milliliters (mL)) should be available to the end-user for a QV task in the form of a DICOM structured report (SR) element.

### Rationale for nnU-net backbone use case

Automated precision diagnostics in cross-sectional imaging of the torso typically require multiscale deep learning (DL) solutions to address complex and heterogeneous pathology with highly variable volumes. DL has demonstrated promising performance on a large variety of medical image analysis tasks (12), however computer vision solutions for torso imaging have been late-comers due to challenges including small target to volume ratios and the highly variable size, appearance, and distribution of pathology (13). A variety of bespoke solutions have been employed for multiscale problems, including coarse-to-fine approaches, and dilated convolutional neural networks with attention modules (14–17).

In 2021, Isensee et al., introduced nnU-net (18), which uses a simple existing u-net backbone and systematizes design choices (including pre-processing, hyperparameter selection, and post-processing) based on the “data fingerprint” of the task at hand, a representation that considers voxel spacing, image size, class ratios, and other dataset-specific features derived from 53 different segmentation tasks. The premise of nnU-net is that such design choices are a more important condition of high performance than architectural modifications, hence the name “no new U-net.” The method achieved state-of-the-art or performance on 23 public datasets in the Isensee et al., paper, and given its ease of implementation and robust performance for a wide variety of tasks, represents a watershed for out-of-the-box automated medical image segmentation. Given the ease of training nnU-net and its high performance, it is now widely used by many investigators. We employed nnU-net in our pipeline due to the low complexity and easy out-of-the-box deployment.

Doran et al. (10) integrated the Open Health Imaging Foundation (OHIF) viewer with the XNAT informatics software platform for quantitative imaging research based on the DICOM web protocol, with advanced features including paintbrush editing tools and integration with NVIDIA’s AI assisted annotation (AIAA). Similarly, Monai label provides active learning and AIAA functionality and can be combined with robust quantification tools as a 3D slicer plug-in (19). The pipelines can be configured with a variety of segmentation algorithms on the back end, including 3D U-Net, DynU-Net and UNETR. A sliding window patch-based method is typically employed to address local GPU memory limitations in training and inference and represents a dependency requiring user configuration that is handled by nnU-net as part of its default settings. To our knowledge, implementations of these tools with nnU-net on the back-end are not currently publicly available, and user selection of patch-based parameters are required with these frameworks.

With our pipeline, investigators have the agency to easily swap in any segmentation algorithm code, including any pre-and post-processing steps such as the sliding window approach described above. The only proviso is that the algorithm receives NIfTI images as input, and outputs segmentations in the NifTI format, as per standard practice in the investigational setting. Further, while new modules may be developed, the lack of a DICOM structured reporting element containing segmentation volumes with XNAT-OHIF, and lack of out-of-the-box DICOM compatibility with 3D Slicer (20) represented barriers for quantitative visualization in the clinical environment that motivated this work.

### Rationale for traumatic pelvic hematoma use case

Whole Body CT has become the routine diagnostic workhorse for admissions with major trauma (21, 22), with potential associated survival benefit (23), but long interpretation times, ranging from 30 to 87 min remains a major bottleneck that limits rapid surgical decision-making (24, 25). Volumetric measurements of hemorrhage are not feasible at the point of care without automation (26, 27), and a recent scoping review found no commercial CAD tools for this purpose (8). A cross-sectional survey of practitioners in the Emergency/Trauma subspeciality reported a desire on the part of most respondents for automated quantitative visualization tools (28). Bleeding pelvic fractures are a leading cause of morbidity and mortality in trauma patients. Once we achieved high saliency visual results that correlated with patient outcomes (14), and further improved DSC using nnU-net, Shadow testing in the clinical environment became desirable for this task. CT volumetry has myriad applications beyond our use case— including objective assessment of malignancy progression with a higher level of precision compared to two-dimensional RECIST criteria (29–31); for measuring organ volumes and body composition parameters (32, 33); and for a wide variety of other applications.

### Purpose

To meet the needs of the community of researchers in this domain, following FAIR (findable, accessible, interoperable, and reusable) principles, we aimed to construct and disseminate a simple and secure, modular, open-source and vendor-agnostic PACS-integrated and DICOM compatible pipeline for end-to-end automated CT quantitative visualization suitable for nnU-net or any other segmentation algorithm. The feasibility of our approach for real-time shadow evaluation in the clinical setting was assessed using clock times for a cascaded nnU-net traumatic pelvic hematoma use case.

## Materials and methods

### Software architecture

**Figure 1:**
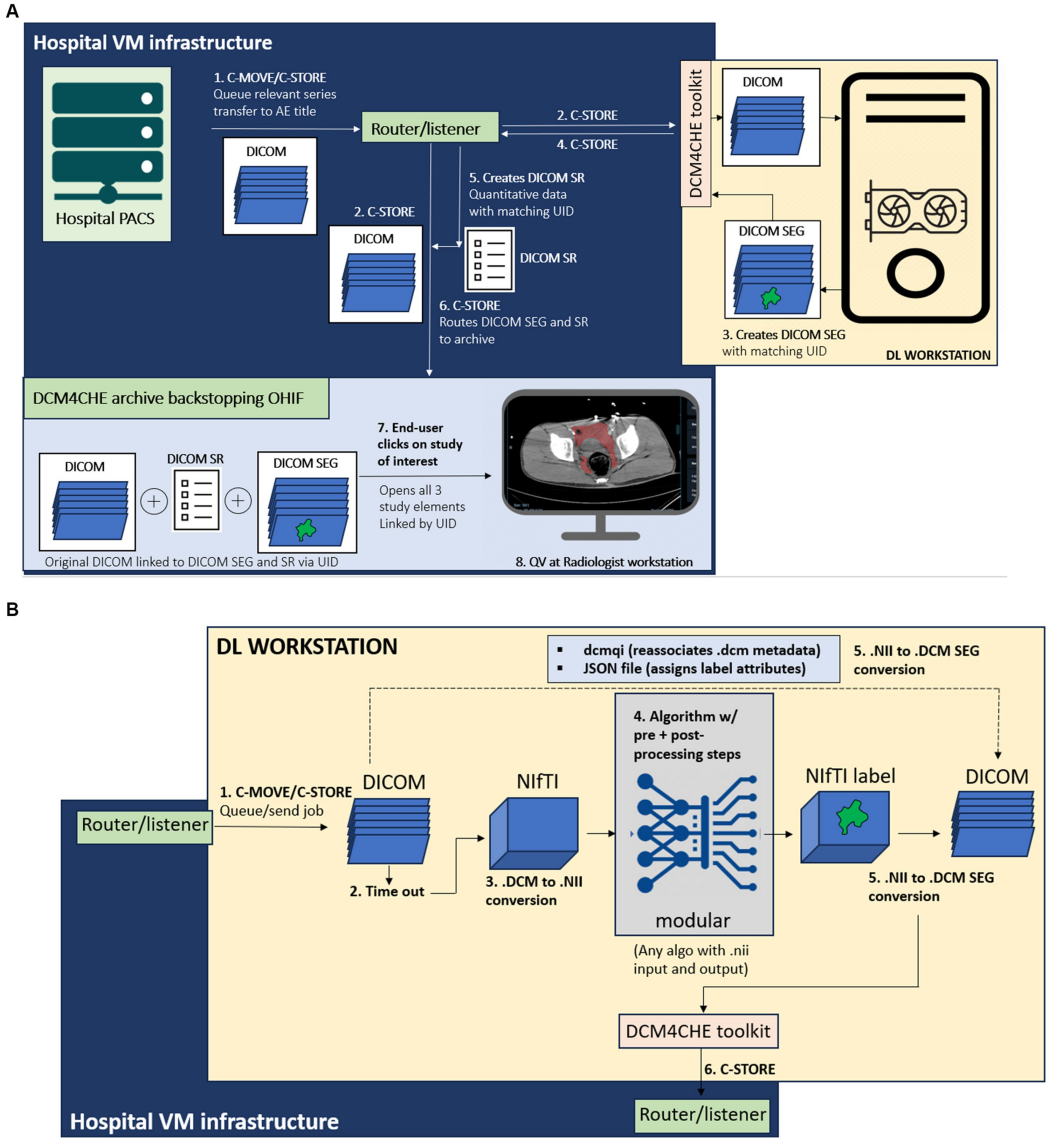
**(A)** Diagram of the overall end-to-end workflow, containerized as software with documentation available through: https://github.com/vastc/. Steps are summarized as follows: In step 1, the study is pushed by a radiologist end-user from a dropdown menu in the radiology PACS to the DICOM router/listener node, which filters the series and performs DICOM-standard anonymization. On the back-end, PACS performs C-MOVE/C-STORE operations, queuing the study for transfer the listener/router application entity (AE) title filtered by pre-specified series descriptions that can be modified per user needs. In step 2, the router performs a C-STORE operation, transferring to (i) the DCM4CHE archive backing the Open Health Imaging Foundation (OHIF) viewer, and (ii) the deep learning (DL) workstation host. The router and viewer run on hospital virtual machine (VM) infrastructure. On the DL workstation host side [see detailed description in **(B)**], a pipeline processes the DICOM series, and returns a DICOM SEG (segmentation) output (step 3), linked to the original study unique identifier (UID) metadata. This is sent by a DCM4CHE DICOM toolkit (DCM4CHE is a robust library used for many healthcare enterprise production applications and utilities) to the router/listener *via* another C-STORE operation (step 4). In step 5, the router/listener extracts quantitative volumetric data from the DICOM SEG and creates a structured report (DICOM SR) element (shown in Figure 5). The DICOM SEG and SR elements linked to the original DICOM series through the same UID are forwarded to the DCM4CHEE archive backstopping OHIF in step 6. This is visualized as a list of archived studies in OHIF by the radiologist end-user. Double-clicking on the study opens the original series for quantitative visualization (QV), with superimposed segmentation and a clickable SR element containing volumetric data in milliliters (see Figures 3–5). **(B)** DL workstation host flow diagram. Steps are summarized as follows. C-MOVE/C-STORE commands from the listener/router (step 1) trigger a time-out function (step 2). DICOM (.dcm) images are individual files, wherein the imaged volume/series is represented by a.dcm image stack. The time-out is necessary to trigger conversion of all.dcm files into a single NifTI (.nii) volume once all images are received (i.e., no further images are sent during the timeout period, set to 30 s). This triggers step 3, DICOM to NIfTI conversion, followed by step 4, image processing by algorithm. We employ nnU-net due to widespread adoption and state-of-the-art performance, but this can be swapped out by users with any segmentation algorithm (with relevant pre-and post-processing steps) that employs NifTI input and output, as per standard practice for segmentation methods in medical imaging analysis. In step 5, the NIfTI label output is converted back to a DICOM SEG series. Metadata is preserved using the dcmqi library and configured. Json file (illustrated in Figure 2). In step 6, the DICOM SEG is sent back to the router/listener for creation of the SR element and routing to the OHIF viewer archive for quantitative visualization as described in this figure.

In the proposed python-based client–server architecture, the study is pushed by an end user from a picture archiving and communications system (PACS) to a DICOM listener/router host where the DICOM series of interest is filtered, anonymized, and sent to (1) a DCM4CHEE query/retrieve archive associated with a zero-footprint Open Health Imaging Foundation (OHIF) DICOM web viewer running on a radiologist workstation, and separately to (2) a deep learning workstation host, where the DICOM series is converted to a NIfTI volume and processed by the DL segmentation algorithm. On this host, the output NIfTI segmentation mask is converted to a DICOM SEG object with a unique identifier (UID) linking to the original DICOM and sent back to the listener/router where the pixel data is used to create a DICOM structured report (SR) element with volumetric information. The DICOM SEG and SR are then routed to the DCM4CHEE query/retrieve archive for secure permission-based quantitative visualization using the OHIF viewer on a radiologist workstation, or other secure Windows-based desktop or laptop. nnU-net is used in our publicly available containerized software. The workflow for the overall pipeline and the deep learning host are illustrated in Figures 1A,B, respectively.

### Building blocks for data transfer and handling: listener/router, archive, and viewer

1. When an asynchronous request is made in PACS to send a study for processing and viewing (by selecting the listener/router node from a dropdown menu), PACS performs a C-STORE operation, which queues the study for transfer inbound to the router Application Entity (AE) Title. The listener/router includes a facility to filter for config file-specified series. The study is anonymized according to the DICOM standard and queued up through its handler using C-STORE for transfer to two AE Titles-a DCM4CHEE archive^1^ and an on-premises deep learning (DL) workstation host. The bespoke listener/router script utilizes the pynetdicom library and is constructed using our own high-level logic to fit the required task.
2. The DCM4CHEE archive provides web services to the OHIF web viewer for retrieving data using the Java-based Web Access to DICOM Objects-RESTful services (WADO-RS) protocol. In short, the DCM4CHEE archive backs the OHIF viewer as the source of DICOM data storage and appears as a list of studies for permission-based viewing by the end-user. Use of a web viewer distinct from PACS is intended to prevent research-grade results from entering the patient’s medical record. The PACS, the router, the DCM4CHEE archive, and the viewer all reside within virtual machine (VM) infrastructure running under the institution’s secure intranet.
3. The deep learning (DL) workstation host is assigned a specific IP address, AE Title, and port. The listener/router executes a C-STORE to the DL host AE Title using the pynetdicom library and has a time-out function that triggers a callback to the DL host to indicate that transfer of all DICOM images from the series of interest is completed. The time out begins with a C-MOVE operation to queue the job up and is followed by a C-STORE operation to a unique directory on the DL host workstation once the time out (from the last time the object of the DICOM series was received) completes, ensuring that NIfTI conversion does not occur prematurely. The callback, which is serialized and currently processed on a single thread, contingent on receipt of the DICOM SEG object to prevent concurrent processing, then triggers code for a series of DL host side conversion and image processing steps described in detail in the next subsection.
4. Once image processing steps are complete, the DL workstation host returns a DICOM SEG object to the listener/router host AE Title. A handler from the listener/router then creates a DICOM structured report (SR) element with a volume value in milliliters extracted from the DICOM SEG pixel data, and executes another C-STORE operation, transferring both SEG and SR elements to the DCM4CHEE archive. A tree of DICOM Unique identifier (UID) study, series, and image DICOM tags associate with the original DICOM series for quantitative visualization in the OHIF web viewer.

### Building blocks for DL host: image format conversion and image processing

The on-premises DL host code runs on a Linux operating system (Ubuntu 20.04; Canonical, London, England) on an AMD Ryzen Threadripper PRO 5965WX 24-Cores CPU workstation with 128 GB of memory, and an NVIDIA GeForce RTX 3090 Ti GPU. The workstation operates within the intranet of the hospital’s radiology department to communicate securely with the listener/router. We used the PyTorch open-source machine learning framework to run nnU-net in inference on this workstation. The components are containerized using Docker and include 1. A DICOM to NIfTI converter that executes upon completion of DICOM transfer, 2. code for cascaded nnU-net inference, and 3. A NIfTI to DICOM SEG converter (Figure 1B). A serialized model from an open-source task (spleen segmentation) is provided in our GitHub repository due to institutional restrictions on our trauma CT data.

**Figure 2:**
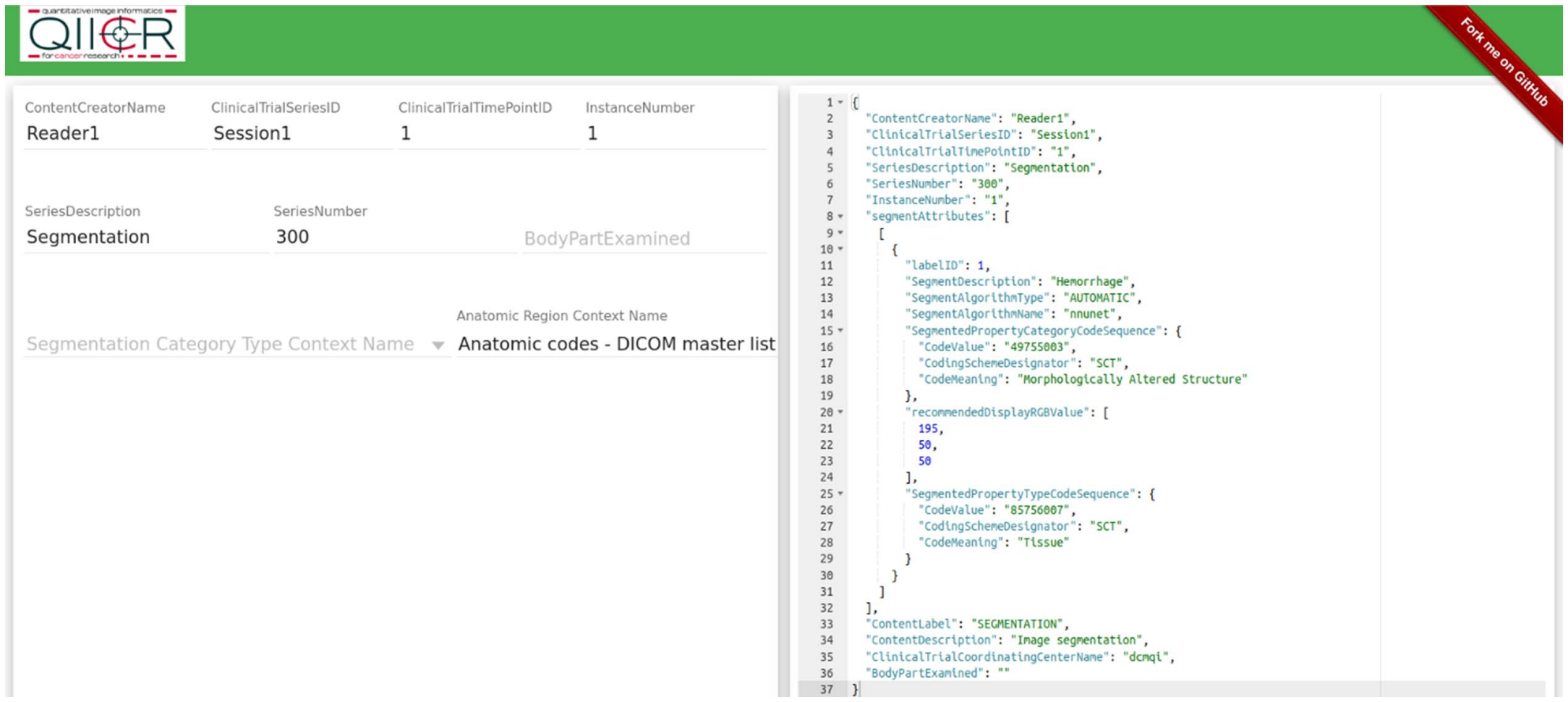
Creating a JSON file for NIfTI to DICOM conversion using dcmqi.

1. DICOM to NIfTI conversion. A listener script written in LINUX sends a command prompt to convert the collected DICOM files to a NIfTI volume after an adjustable delay time set to 30 s. The DICOM to NIfTI converter is implemented using the DICOM2nifti library.^2^ This building block is used to convert the DICOM sequence to a.nii file. After the data conversion is completed, the script executes model inference.
2. Trained model. Prior to deployment in our pipeline, all pre-processing, training, and post-processing steps were completed by nn-Unet in five-fold cross-validation per specifications of this self-configuring method (18). We employed the 3D cascaded low-resolution to high-resolution nnU-net architecture and model which gives state of the art performance for multiscale segmentation tasks. The pipeline can be reconfigured with other networks in place of nnU-net to suit investigators’ needs.
3. The segmentation output is converted from NIfTI to a DICOM SEG object linked to the original segmentation with a UID using dcmqi (34) (DICOM for Quantitative Imaging) library^3^ and dcmqi-created JSON file (Figure 2), which also specifies target attributes such as “hemorrhage,” pelvic hematoma, and the color of the mask or contour. Once created, the script calls a DCM4CHE toolkit installed on the DL host to perform a C-STORE operation, transferring the DICOM SEG object back to the router/listener discussed in the previous subheading.

### Patient dataset

Performance of pelvic hematoma segmentation in 5-fold cross-validation on 253 training cross-sectional CT studies with over 100,000 2D images is previously reported (15) with DSC improving further from 0.71 to 0.75 with cascaded nnU-net. A convenience sample of 21 new, unseen patients with pelvic hematoma between 1/1/2018 and 2/1/2021 was used to record clock times (described below). Patients had a mean age of 42.1 years (range: 21–82) and were 62% male. 20 had blunt injury mechanisms including motor vehicle (*n* = 7), motorcycle (*n* = 3), and bicycle collision (*n* = 1), pedestrian struck by car (*n* = 5), fall (*n* = 3), and industrial crush injury (*n* = 1) = and 1 patient had a pelvic gunshot wound. Trauma WBCTs at our institution are performed on either of two trauma bay-adjacent scanners-a 64 section unit (Brilliance; Philips Healthcare, Andover, Mass) or a dual source 128-section scanner (Siemens Force; Siemens, Erlangen, Germany), using 100 mL of intravenous contrast material (Omnipaque [iohexol; 350 mg of iodine per milliliter]; GE Healthcare, Chicago, IL). Arterial phase images are obtained from the thoracic inlet through the greater trochanters, and portal venous (PV) phase images starting at the dome of the diaphragm. Studies are archived with 3 mm section thickness. The number of PVP axial images for a given study ranged from 91 to 203.

### Clock times

To test our software pipeline, the 21 consecutive cases were pushed from PACS. Total clock times from the beginning of the PACS C-STORE operation to completion of transfer to the viewer were recorded, along with times for the following steps: (1) clock times for model inference, (2) clock times for all data conversion and transfer steps on the DL-host side, and (3) combined clock times for virtual and on-premises host steps without nnU-net inference.

## Results

The software is available on our github repository,^4^ with relevant links to our customizable listener/router docker container, DL host docker container, other components (DCM4CHEE container and OHIF viewer) and config files for modifiable site-specific configuration of the listener/router (e.g., file names for filtering, time-out delay, AE titles, IP addresses, and ports). readme files are provided for documentation.

**Figure 3:**
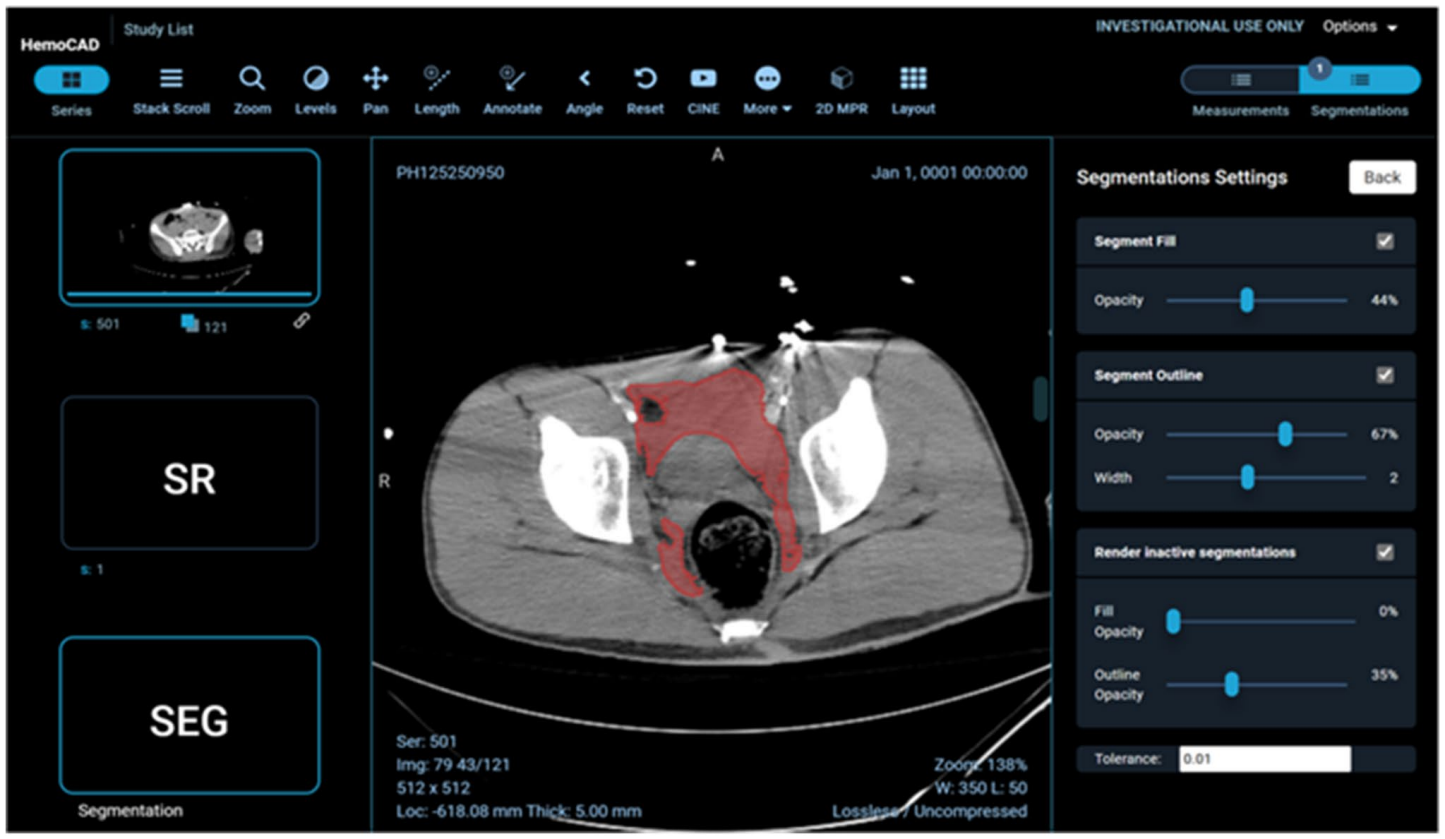
Client-side display of DICOM images, segmentation mask, and a structured report element including pelvic hematoma volumes in mL.

**Figure 4:**
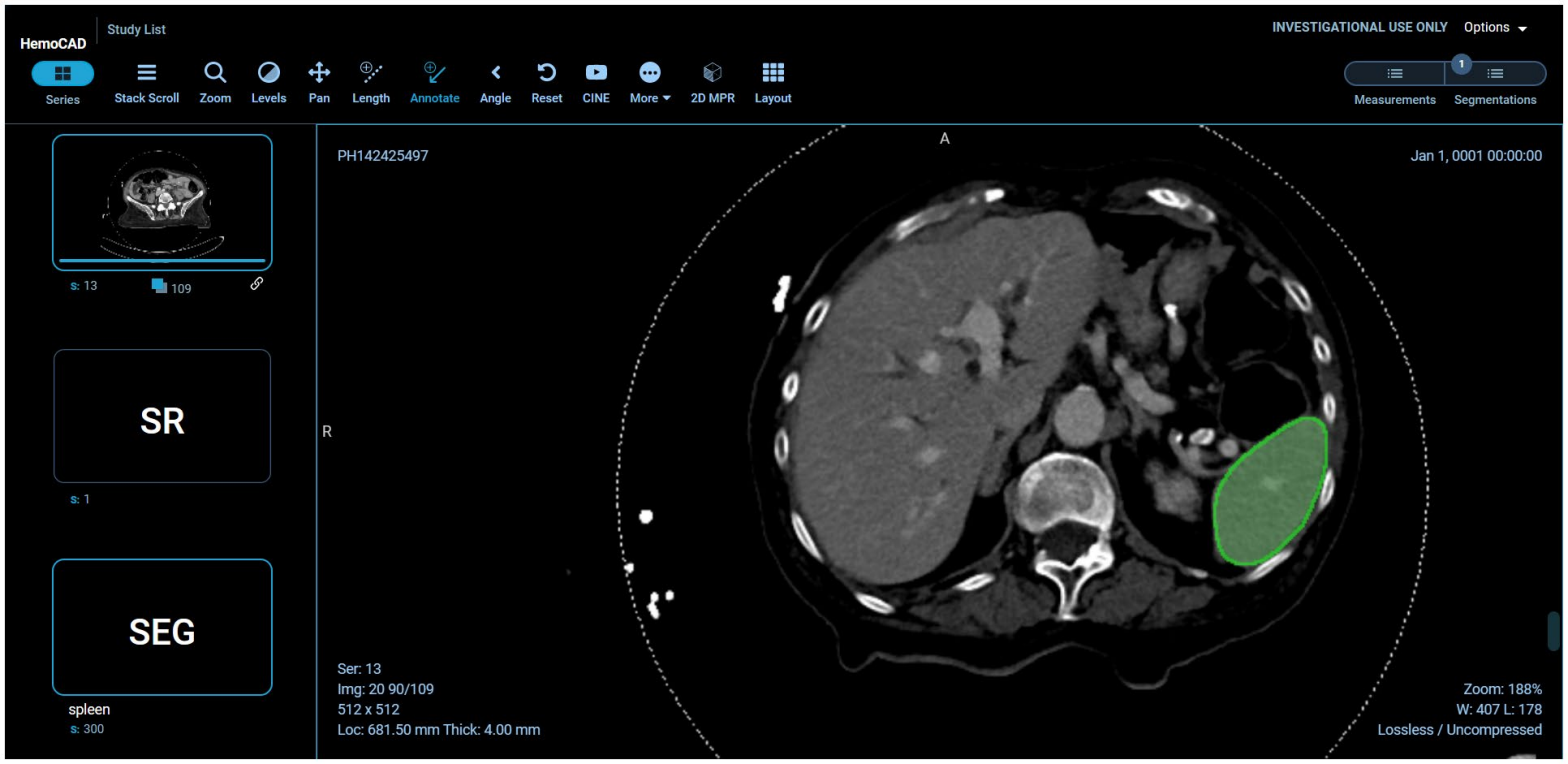
Interoperability using various models on the back-end. In this case, the pelvic hematoma model was swapped out for a public model trained on the spleen segmentation dataset (task_009 spleen) from the public nnU-net repository (https://zenodo.org/record/3734294).

**Figure 5:**
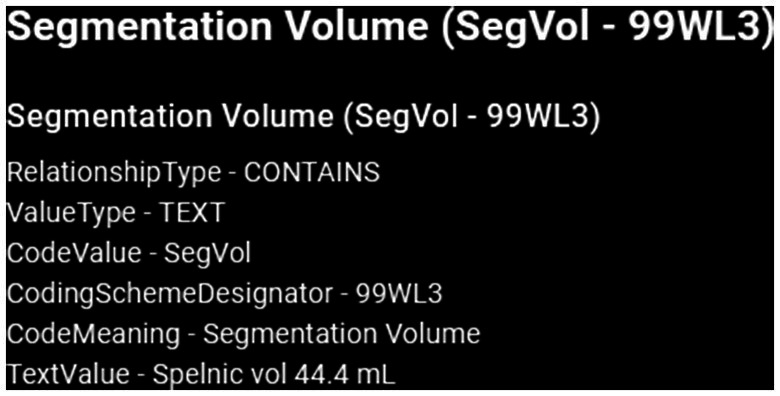
SR element corresponding with this figure. Structure/pathology type and color scheme is derived from the original JSON file. Pixel data is extracted from the SEG study instance unique identifier (UID). In future iterations, SR element meta-data will be used to auto populate radiology reports with statements such as “splenic vol: 44.4 mL” Radiologists find this a highly desirable functionality (28).

Example visual results using the OHIF viewer (35) with the DICOM SEG mask overlaid on the linked anonymized DICOM series are shown (for pelvic hematoma segmentation, see Figure 3; for splenic segmentation, see Figure 4). A modular structured report element with a statement of “Splenic volume: 40 mL” is shown in Figure 5.

Returning to pelvic hematoma, Mean total clock time in the 21 patients from PACS send request to completion of receipt of the DICOM SEG object and structured report in the DCM4CHEE archive was 5 min 32 s (± SD of 1 min and 26 s; min: 3 min 16 s, max: 9 min, 2 s). nnU-net inference times contributed to over 89% of the total time. Mean clock time for all other on-premises DL host steps totaled only 5.4 s (± SD: 1.3 s: min: 2.0 s; max: 7.0 s). Excluding inference time, mean combined total clock time for all steps on the listener/router virtual infrastructure side and DL host side of the proposed software platform was 38.5 s (± SD of 4.7 s) (Table 1). Pelvic hematoma volumes ranged from 28.0 to 924.7 mL (median: 301.5 mL, IQR [94,8, 491.7]). Pearson correlation *r* values between volumes and nnU-net inference times (*r* = 0.12 *p* = 0.61), between volumes and total clock times (*r* = 0.06, *p* = 0.8) and between the number of slices per DICOM series and clock times (*r* = 0.17, *p* = 0.5) were all poor, such that these factors had no discernable effect on processing times. DICOM SEG volumes corresponded exactly (to the nearest 1/10th of a milliliter) to those obtained from NIfTI volumes using the 3D slicer image computing platform quantification module.^5^ Interoperability with the public spleen model is illustrated in Figure 4.

**Table 1:** Summary of clock times.

	Total time	Inference time	Other DL host-side steps	Virtual and on-premises host steps (w/o inference)
Mean	332.4	293.9	5.4	38.5
Median	337.6	301.0	5.5	36.3
std dev	86.1	85.3	1.3	4.7
1st quartile	261.1	227.0	5.0	35.3
3rd quartile	345.5	307.0	6.0	42.5
Min	195.9	161.0	2.0	34.1
Max	541.8	507.0	7.0	48.1

Total time = time between PACS send operation and images received in client-side OHIF reviewer. All times presented in seconds and rounded to the nearest 10th of a second. Std dev, standard deviation; DL, deep learning; w/o, without; Min, minimum; Max, maximum.

## Discussion

There is a need for open-source software that integrates AI/ML algorithms into clinical workflows for pre-clinical evaluation (6). Jansen et al. (9) developed a vendor-agnostic platform for integrating AI applications into digital pathology infrastructures, and Sohn et al. (13) introduced a vendor-agnostic platform for integrating AI into radiology infrastructures using breast density classification on 2D mammography as a use case. XNAT-OHIF integrates with DICOM (4) but to our knowledge and based on personal correspondence, did not provide quantitative volumetric information. Segmentation and quantification of pathology— such as advanced malignancy (14), lung nodule size (15), or COVID infiltrate volume (16) has generated considerable interest as potential precision medicine tools since manual segmentations are not feasible at the point of care, and there is considerable information loss and subjectivity associated with diameter-based measurements (17).

In this work, we address an unmet need for tools that integrate automated cross-sectional imaging segmentation results into a DICOM-based quantitative visualization clinical workflow. Since its introduction in 2021 (3), nnU-net has emerged as a widely-employed robust and easy to train method for segmentation tasks in medical imaging in the NIfTI format. Our containerized open-source vendor-agnostic software is intended for clinical-translational researchers who wish to deploy their segmentation models in inference for further testing on new cases encountered in the clinical workflow using the DICOM standard. For those wishing to use cascaded nnU-net, the pipeline can be used out-of-the-box with relevant.pkl files.

On the virtual infrastructure host side, a router/listener anonymizes and handles DICOM series which are sent to a DICOM query/retrieve archive backing an OHIF web viewer, and to an on-premises single GPU-based DL workstation. On the DL host side, DICOM series are converted to NIfTI and processed by the segmentation algorithm. A NIfTI segmentation mask sharing the same UID as the DICOM files is converted to a DICOM SEG object and returned to the router/listener where a DICOM SR element containing segmentation volume (in mL) is created. The DICOM SEG and SR objects are then sent to the DICOM archive for viewing. The segmentation and quantitative information are thereby harmonized to the same format as the original DICOM data. The building blocks were implemented using publicly available open-source libraries, which made our software vendor-agnostic and easily deployable, along FAIR principles. By open-sourcing the proposed software, we encourage radiologists and radiology IT developers to integrate more data transfer functionality and more models into the clinical radiology workflow.

Radiologists should be able to receive verifiable quantitative results well within CT report turnaround times should they wish, for example, to include this information in their reports within the framework of a prospective research study. We tested the software using 21 consecutive patients with traumatic pelvic hematoma. Clinical interpretation of WBCT scans for polytrauma or cancer staging typically exceeds 30 min, and results were available within a fraction of this minimum expected turnaround time in all cases.

Using our method, we achieved a mean clock time of 5 min and 32 s using a workstation with a single NVIDIA GeForce RTX 3090 Ti graphics card. This is approximately 1/5th of a typical report turnaround time for a patient undergoing WBCT for suspected polytrauma. nnU-net inference is responsible for over 89% of the clock time, and the time for all other on-premises DL host-side and virtual router/listener-side steps were found to be negligible, with a mean of only 38.5 s (which includes the 30 s time-out). Therefore, investigators can expect minimal delays resulting from data transfer within the pipeline itself, with clock times dependent almost wholly on algorithm inference. Given lack of correlation with number of slices or wide range of target volumes using nn-Unet, we speculate that slice thickness (whether 5 mm or < 1 mm) or the volume of pathology (whether for example, adrenal nodules, tiny pancreatic cysts, liver masses, or widely metastatic disease) will have little impact on send-to-receive times.

There are limitations to our pilot study. We describe clock times for 21 patients on a single task. However, any algorithm or model can be used. We include a publicly available nnU-net model for spleen segmentation (pretrained nnU-net model Task009_Spleen) in our GitHub link to initially operationalize the deployed pipeline. In the future, end-users may wish to have an “always-on” system that sends the series of interest for every patient directly from a scanner AE Title. Given the lag time associated with post-processing, study completion by the technologist, and transfer from the scanner to PACS, sending a given series from the scanner on creation could result in substantial time savings, however this may not be desirable without an initial rapid detection or classification step to separate positive from negative studies for a given feature of interest. To this end, we have recently developed a message broker and pop-up notification tool for our pipeline and plan to release these in future updates. Sending a study from PACS to the listener/router node selected from a drop-down menu is currently the only manual step. To simplify the process, we are working on an integrated PACS icon. We are also exploring solutions for auto-population of quantitative results in radiology reports. Our method currently employs nnU-net and investigators wishing to implement other segmentation algorithms and models that use the NifTI format as input and output (as is standard for segmentation tasks in medical imaging analysis) will need to simply swap out the code and models.

## Conclusion

In conclusion, we have developed and released a simple open-source vendor-agnostic PACS and DICOM compatible software package for investigators wishing to shadow test volumetry-based algorithms in the clinical environment. The method approximates FDA-designated IPQ or CADx quantitative volumetry-based CAD tools and is meant to accelerate deployment of precision medicine-based applications for cross-sectional imaging.

## Data availability statement

The code presented in this study can be found in online repositories. The name of the repository can be found here: https://github.com/vastc/.

## Ethics statement

The studies involving humans were approved by University of Maryland Institutional Review Board. The studies were conducted in accordance with the local legislation and institutional requirements. The ethics committee/institutional review board waived the requirement of written informed consent for participation from the participants or the participants’ legal guardians/next of kin because Category 4 exemption-deidentified data. Written informed consent was not obtained from the individual(s) for the publication of any potentially identifiable images or data included in this article because Category 4 exemption-deidentified data.

## Author contributions

WL, LZ, DD, MU, HC, and JH: concepts and design. GL and DD: data acquisition. DD and LZ: data analysis, interpretation, original draft preparation, and literature research. DD: statistical analysis and funding acquisition. LZ, DD, WL, and MU: manuscript review, editing, and revision for important intellectual content. All authors contributed to the article and approved the submitted version.

## Footnotes

^1^DCM4CHE library: https://www.dcm4che.org/.

^2^https://github.com/icometrix/DICOM2nifti

^3^https://github.com/qiicr/dcmqi

^4^https://github.com/vastc/

^5^www.slicer.org, version 5.0.3.
